# Endoplasmic reticulum stress promotes the release of exosomal PD-L1 from head and neck cancer cells and facilitates M2 macrophage polarization

**DOI:** 10.1186/s12964-021-00810-2

**Published:** 2022-01-28

**Authors:** Yi Yuan, Pengfei Jiao, Zeyu Wang, Mengqi Chen, Hongming Du, Liang Xu, Juanyong Xu, Youjin Dai, Fu-gen Wu, Yaqin Zhang, Heming Wu

**Affiliations:** 1 Jiangsu Key Laboratory of Oral Diseases, Nanjing Medical University, Nanjing, 210029 People’s Republic of China; 2 Department of Oral and Maxillofacial Surgery, Affiliated Hospital of Stomatology, Nanjing Medical University, Nanjing, 210029 People’s Republic of China; 3 Key Laboratory of Model Animal Research, Animal Core Facility of Nanjing Medical University, Nanjing Medical University, Nanjing, 211166 Jiangsu People’s Republic of China; 4 State Key Laboratory of Bioelectronics, School of Biological Science and Medical Engineering, Southeast University, Nanjing, 210096 Jiangsu People’s Republic of China; 5 Key Laboratory of Human Functional Genomics of Jiangsu Province, Department of Biochemistry and Molecular Biology, Nanjing Medical University, Nanjing, 211166 Jiangsu People’s Republic of China

**Keywords:** Oral squamous cell carcinoma, Macrophage, Endoplasmic reticulum stress, Exosome, PD-L1

## Abstract

**Background:**

Endoplasmic reticulum (ER) stress has been found to foster the escape of cancer cells from immune surveillance and upregulate PD-L1 expression. However, the underlying mechanisms are unknown.

**Methods:**

While analyzing the protein levels using immunofluorescence and Western blotting, the RNA levels were measured using qRT-PCR. Ten injection of exosomes into six-week-old nude mice was made through the tail vein once every other day in total.

**Results:**

The expression of certain ER stress markers such as PERK (PKR-like endoplasmic reticulum kinase), ATF6 (activating transcription factor 6), and GRP78 (glucose-regulated protein 78), was found to be upregulated in the oral squamous cell carcinoma (OSCC) tissues and related to poor overall survival. There is a positive relationship between the extent of ER stress-related proteins and a cluster of PD-L1 expression and macrophage infiltration among the OSCC tissues. Further, incubation with exosomes derived from ER-stressed HN4 cells (Exo-ER) was found to upregulate PD-L1 extents in macrophages in vitro and in vivo, and macrophage polarization toward the M2 subtype was promoted by upregulating PD-L1.

**Conclusions:**

ER stress causes OSCC cells to secrete exosomal PD-L1 and upregulates PD-L1 expression in macrophages to drive M2 macrophage polarization. The delineation of a new exosome-modulated mechanism was made for OSCC–macrophage crosstalk driving tumor development and to be examined for its therapeutic use.

**Graphical abstract:**

Exosomal PD-L1 secreted by ER-stressed OSCC cells promoted M2 macrophage polarization. 
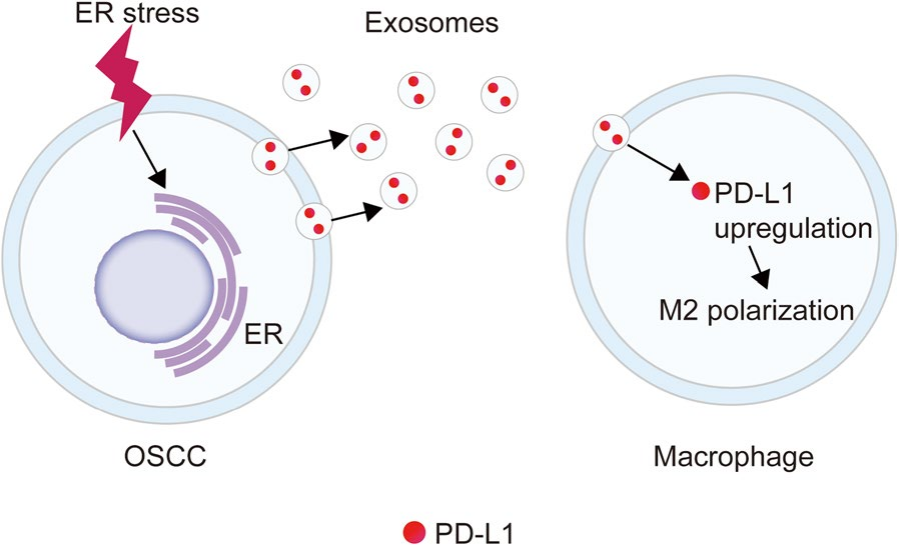

**Video Abstract**.

**Supplementary Information:**

The online version contains supplementary material available at 10.1186/s12964-021-00810-2.

## Background

Oral squamous cell carcinoma (OSCC) is the most usual kind of cancer of the head and neck area. Despite the recent progress in knowing the diagnosis, molecular biology, and treating OSCC, the associated five-year survival rate has been less than 50% for the past 30 years—primarily because of cases of metastasis or local incontrollable recurrence [1]. OSCC is mainly caused by tobacco use (chewing and smoking). Other risk factors include infections with certain types of human papillomavirus, certain workplace exposures, and radiation exposure. The diagnosis is confirmed using a tissue biopsy, with computed tomography and blood tests often being used to check the degree of spread. Traditional treatments include surgery, chemotherapy, and radiation therapy [2, 3].

While the immune system does participate in anticancer activity, it took part in developing and advancing cancer [4]. The immunosuppressive status of OSCC has attracted an increasing amount of attention in the research, and the effectiveness of immune checkpoint blockades and the overexpression of immune checkpoint molecules for OSCC therapy has been confirmed [5]. Tumor cells remodel their surrounding cells to advance cancers, tumorigenesis, and the invasion of adjacent tissues in the tumor-associated environment (TME) [6]. Further, 5–40% of the mass of solid tumors consists of macrophages [7–9]. The involvement of macrophages is now established and is starting to be better understood. Macrophages serve as an interface between innate and acquired immunity, and they become polarized into M1 and M2 phenotypes depending on the expression of the cytokines, receptors, and effector molecules [10]. Under physiological situations, the polarizatioon of macrophages into the proinflammatory and antitumor M1 phenotype was made; nevertheless, tumor cells can induce macrophages to switch to activated alternatively the M2 phenotype via some pathways (CCL-2, IL-4, IL-6, IL-8, IL-10, TGF-β, and PD-1/PD-L1 [programmed cell death-ligand 1]). The M2 macrophages secrete high extents of cytokines, chemokines, enzymes, and growth factors including VEGF, PDGF, TGF-β, and FGF, and some matrix metalloproteinases. These upregulate inflammation while simultaneously promoting immunosuppression, angiogenesis, migration, tumor progression, metastasis, and treatment resistance [10, 11].

Tumor progression also responds to endoplasmic reticulum (ER) stress, which is a vital cellular response that maintains cell survival through the activation of the unfolded protein response. ER stress acts as a point of “protein quality control” in cells and facilitates several cellular functions, including protein folding and Ca2+ homeostasis, by processing nascent membrane and secretory proteins in a Ca2+-dependent way [12]. ER stress is controlled by several associated proteins, including protein kinase R–like ER kinase (PERK), activating transcription factor 6 (ATF6), and glucose-regulated protein 78 (GRP78). Different tumors have reported the initiation of ER stress, and promoted further tumor progression [13, 14]. ER stress induces tumor cell escape from immunological surveillance, and its activation in immune cells affects the role of infiltrating immune cells. For instance, ER stress increases a range of inflammatory factors, including interleukin (IL)-23 and IL-6, in macrophages [15]. Further, ER-stressed tumor cells also modify immune cell functions through the release of ER stress-associated molecules and, subsequently, promoting tumor survival, progress, and metastasis [16]. However, the mechanisms by which ER-stressed tumor cells cultivate immune cells and suppress immune responses remain unknown.

One possible mechanism involves exosomes, which are 30–200 nm membrane vesicles that are involved in cell-to-cell communication. Exosomes are loaded with DNA, proteins, and coding and non-coding RNAs and are released from living cells into the extracellular environment. The exosomes derived from cancer cells vary from those secreted by normal cells [17], and the distinctive differences in exosome content in certain cancer cells can be adopted as diagnostic or prognostic markers. For example, miR-21 is a famous oncomir that can be adopted as a diagnostic marker in ovarian cancer [18]. The exosomes released by cancer cells interact with myeloid-derived suppressor cells (MDSCs), tumor-related macrophages (TAMs), or tumor-infiltrating T cells (TILs). They cause a phenotypic change of stroma cells and tumor-infiltrating immune cells, thereby generating a tumor-permissive microenvironment. It was shown that PD-L1 proteins are packaged in exosomes purified, which indicates that these vesicles may deliver protein information to recipient cells [19]. These findings suggest that PD-L1 is one of the vital molecules taking part in exosome-mediated intercellular communication.

However, only a few researches have investigated whether ER stress influences the transfer of exosomal PD-L1 and whether exosomal PD-L1 affects OSCC tumor progression. Therefore, the current research aimed to examine whether ER-stressed OSCC cells can transmit PD-L1-enriched exosomes to macrophages and whether the polarization of macrophages are modulated by these exosomal PD-L1.

## Methods

### Patients and samples

Permitted by the ethics committee of the Stomatology Hospital of Nanjing Medical University (Nanjing, China), the current protocol accords with the ethical guidelines of the 1975 Declaration of Helsinki. All participating patients in accordance with the ethical principles of the Declaration of Helsinki submitted the written informed consents. 100 OSCC patients who had been admitted to the Stomatology Hospital of Nanjing Medical University between 2019 and 2020 provided primary OSCC tumor tissues. The collection of tumor samples was made during the surgery, and the cutting of non-tumor samples to at least 5 cm was conducted. Tumours were categorised on basis of the World Health Organization (WHO) classification and the International Union Against Cancer tumour-node-metastasis (TNM) classification system. The OSCC tumor tissues were formalin-fixed and paraffin-embedded for histopathological diagnosis and the establishment of paraffin-embedded tissue staining.

### Cell culture and stimulation

Human head and neck squamous cell carcinoma cell line HN4 was acquired from the research laboratory of the Stomatology Hospital of Nanjing Medical University (Nanjing, China). A multiplex polymerase chain reaction (PCR) was employed to verify the cell lines. The culture of HN4 cells was performed in DMEM F-12 (Gibco, Cat No: 11320033), which was supplemented with 10% fetal bovine serum (Gibco, Cat No: 16000036) under 5% CO_2_ atmosphere at 37 °C. The culture medium included penicillin (100 U/ml) and streptomycin (100 mg/ml) (Invitrogen, 15140-122). Next, 1 × 10^5^ HN4 cells were planted per dish in 100-mm cell culture dishes and passaged every two or three days. To obtain ER-stressed HN4 cells, the cells were treated with IFN-γ (500 U/mL) for 48 h. In addition, human monocytic THP-1 cells were kept in standard RPMI 1640 medium added with 10 % heat inactivated fetal bovine serum (Invitrogen) and added with 10 mM Hepes (Gibco, Cat No: #15630-056), 1 mM pyruvate (Gibco, Cat No: #11360-039), 2.5 g/l D-glucose (Merck) and 50 pM ß-mercaptoethanol (Gibco; Cat No: 31350–010) in a 5 % CO2, water-saturated atmosphere. The differentiation of THP-1 monocytes into macrophages was made by 24-hour incubation with 150 nM phorbol 12-myristate 13-acetate (PMA, Sigma, Cat No: P8139) added in RPMI medium.

### Cell transfection

The sequence of the siRNA oligo against PD-L1 (siPD-L1; MW = 13,788.9 g/mol) was 5′-ThioMC6-D/GGUCAACGCCACAGCGAAUUU-3′ (sense sequence) and 5′-PAUUCGCUGUGGCGUUGACCUU-3′ (anti-sense sequence). The sequence of the scrambled siRNA oligo (siNC; MW = 13,728.8 g/mol) was 5′-ThioMC6-D/UGGUUUACAUGUCGACUAAUU-3′ (sense sequence) and 5′-PUUAGUCGACAUGUAAACCAUU-3′ (anti-sense sequence). A plasmid DNA which expressed PD-L1 (OE-PD-L1) was purchased from PPL (Public Protein/Plasmid Library, China). The day before transfection, the seeding of HN4 cells was made in cell culture plates containing a complete DMEM F-12 medium and incubated for 24 h. The following day, transfection was performed applying Lipofectamine 2000 (Invitrogen, Cat No: 11668019) on basis of the manufacturer’s instructions.

### Western blot analysis

The extraction of proteins was conducted from human OSCC tumor tissues and paired paracarcinoma tissues and indicated exosomes or macrophages, and measured by Bio-Rad protein assay. The proteins were analyzed using western blotting by separating by the 10% SDS-PAGE, blotting onto a PVDF membrane, blocking with 5% non-fat dry milk (Bio-Rad, Cat No: 1706404) or 5% BSA in a TBS-T buffer (10 mM Tris, 150 mM NaCl, and 0.2% Tween-20 [pH 8.0]). The overnight incubation of membrane was made with a primary antibody at 4 °C and, then, with a secondary antibody conjugated with alkaline phosphatase (for 1 h at room temperature); a chemiluminescence approach was adopted to detect the signal. The primary antibodies below were used: anti-PERK (Abcam, Cat No: ab65142), anti-GRP78 (Abcam, Cat No: ab21685), anti-ATF6 (Abcam, Cat No:ab203119), anti-GAPDH (Abcam, Cat No:ab8245), and anti-PD-L1 (Cell Signaling Technology, Cat No: 13684). The Super Signal West Dura substrate (Thermo Fisher, Cat No: 34075 was adopted to visualize the immunoreactive bands.

### Quantitative RT-PCR

The TRIzol reagent was adopted to isolate the total RNA, and a Superscript VILO cDNA synthesis kit (Life Technologies**,** Cat No: 11754) was employed to synthesize cDNA. Quantitative real‐time PCR experiments were made with the Step‐One PCR System (Applied Biosystems). A real‐time RT‐PCR data analysis was made according to the 2−ΔΔCt method applying the threshold cycle (Ct) values for the target genes and GAPDH as an endogenous control gene. All assays were made in triplicate. The Additional file 1: Table S1 lists the PCR primers.

### Immunohistochemistry

The OSCC samples were analyzed immunohistochemically using antibodies against PERK (Abcam, Cat No: ab65142), ATF6 (Abcam, Cat No: ab203119), or GRP78 (Abcam, Cat No: ab21685). Next, the brief deparaffinization of 4-μm sections was made in xylene, followed by dehydration in an ethanol diluent serial, and 0.3% hydrogen peroxidase was used to inhibit the activity of endogenous peroxidase at room temperature for 30 min. Then, a microwave oven was adopted to perform antigen retrieval before the blocking of non-specific binding with 5% normal goat serum at 37 °C for 15 min. The overnight incubation of sections was performed with the primary antibody at 4 °C in a humidified box. After three washes with PBS, biotinylated anti-mouse immunoglobulin was adopted for the incubation of sections at 37 °C for 30 min. The visualization of samples was made using 3, 3-diaminobenzidine tetrahydrochloride, followed by counterstaining with hematoxylin. For the analysis, the positive percentages of ER stress-related proteins were explored in five random areas of every sample using Image J.

### Immunofluorescence

The sections were processed as per the protocol of immunohistochemistry and incubated with the desired primary antibodies against CD68 (Abcam, Clone KP1), CD163 (Abcam, Clone EDHu-1), CD11c (Abcam, Clone EP1347Y), and PD-L1 (Cell Signaling Technology, Cat No: 13684) at 4 °C overnight. Then, the goat anti-mouse IgG (H + L) AF488 and goat anti-rabbit IgG (H + L) AF555 secondary antibodies were applied for 1 h before counterstaining the nuclei with DAPI (Sigma-Aldrich, Cat No:D8417) for 10 min at 37 °C. Finally, the capture and analysis of figures were made with an Olympus FV1000 laser scanning confocal microscope and the FlowView software at the Flow Cytometry and Cellular Imaging Facility of MD Anderson. The same exposure time was adopted to capture all the images for all samples.

### Exosome isolation and identification

Exosomes from the 48-h culture supernatants of the control or treated HN4 cells were extracted using a total exosome isolation reagent (Invitrogen, Cat No: 4478359). Thawed in a 25 °C water bath, the supernatants were centrifuged at 2000×*g* for 30 min to eliminate any cells and debris. Next, 500 μL of the total exosome separation reagent were put to 1 ml of the supernatants, and the vortex of mixture was performed until it was homogeneous. After overnight incubation at 4 °C, the 60-minute centrifugal of sample was performed at 12000×*g* at room temperature. The disolution of exosome pellet was performed in 100 µL PBS. The isolation of the exosomes from the supernatants was confirmed using transmission electron microscopy (TEM), nanosizer, and zeta potential analyses. The exosomes were fixed in a glutaraldehyde solution of 2.5% for at least 2 h before transferring 10 μL of the diluted mixtures to a cleaned copper grid. Figures were taken by TEM (Jeol) after the mixtures were dyed with a phosphotungstic acid solution of 2%. For the nanosizer analysis, the dilution of separated exosome samples was performed 2000-fold, followed by resuspending in PBS for a size distribution analysis with a Nano series-Nano-ZS zetasizer (Malvern Instruments Ltd.) on basis of the manufacturer’s guidance. The zeta potential of the exosomes diluted in PBS (25 μg/mL) was determined using an ELSZ-DN2 zeta potential analyzer (Otsuka Electronics) on basis of the manufacturer’s instructions.

### THP-1 macrophages engulf exosomes

PKH26, a red fluorescent dye, was used to label the purified exosomes on basis of the manufacturer’s suggestions. Next, the incubation of THP-1 macrophages was performed with the PKH26-labeled exosomes at 37 °C for 12 h, and confocal microscopy (Leica, Germany) was employed to assess their uptake of the exosomes.

### Animal experiments

The animal experiments strictly followed the principles of minimal pain, suffering, and discomfort experienced by the test animals. The Animal Research Committee, Graduate School of Medicine, Nanjing Medical University approved all protocols. Ten injection of exosomes was performed through the tail vein into six-week-old nude mice once every other day. Then, the peritoneal macrophages were separated 12 h after the final injection. The PD-L1 levels were analyzed using western blotting and qRT-PCR. Six mice were adopted per group in the animal experiments.

### Statistical analysis

The data are expressed as mean ± SEM. The statistical diversities between the two groups were decided by the student’s t-test. The diversities observed between various groups were tested applying variance (ANOVA) analysis, and their significance was confirmed applying Fisher’s protected least-significant diversity test. The influence of the prognostic elements on tumor-assoicated survival was evaluated by Kaplan-Meier estimates, and the comparison of subgroups was conducted using the Breslow test for univariate analyses. Further, two-tailed *P* values of 0.05 or less were of statistical significance.

## Results

### ER stress is activated and positively relates to poor survival in OSCC patients

**Fig. 1:**
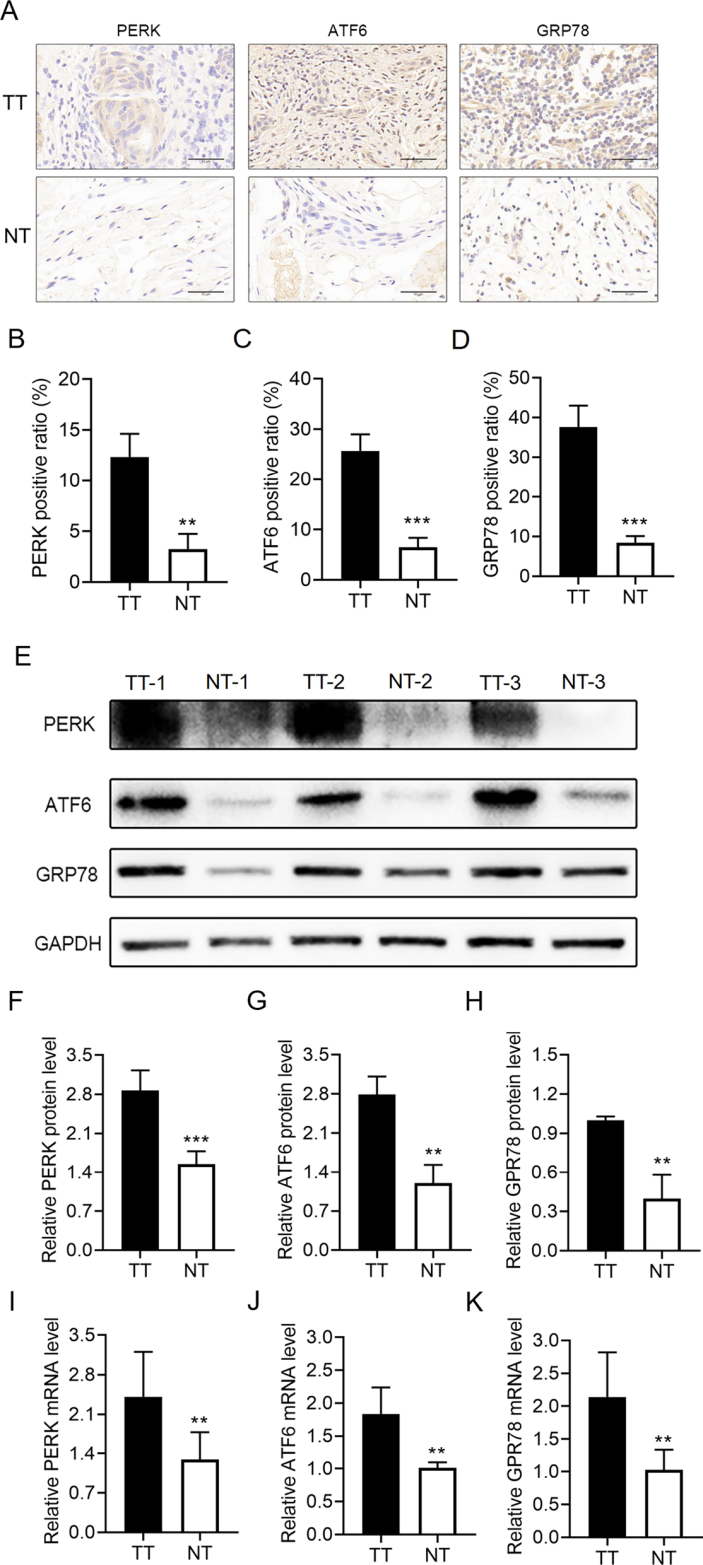
ER stress is activated in the OSCC tissues. **A** Representative immunohistochemical images of the ER stress marker (PERK, ATF6, and GRP78) staining in the paraneoplastic tissues (NT) and OSCC tissues (TT) of patients with OSCC (scale bar = 50 μm) (n = 3). **B**–**D** Quantification of PERK (**B**), ATF6 (**C**), and GRP78-positive (**D**) staining in (**A**) (n = 3). **E** Western blot analyses of PERK, ATF6, and GRP78 protein expression in the paraneoplastic tissues (NT) and OSCC tissues (TT) of patients with OSCC. **F**–**H** Quantification of the protein levels of PERK (**F**), ATF6 (**G**), and GRP78 (**H**) in (**E**). **I**–**K** The level of PERK (**I**), ATF6 (**J**), and GRP78 mRNA (**K**) in the paraneoplastic tissues (NT) and OSCC tissues (TT) of patients with OSCC (n = 3) (**P < 0.01 and ***P < 0.001)

**Fig. 2:**
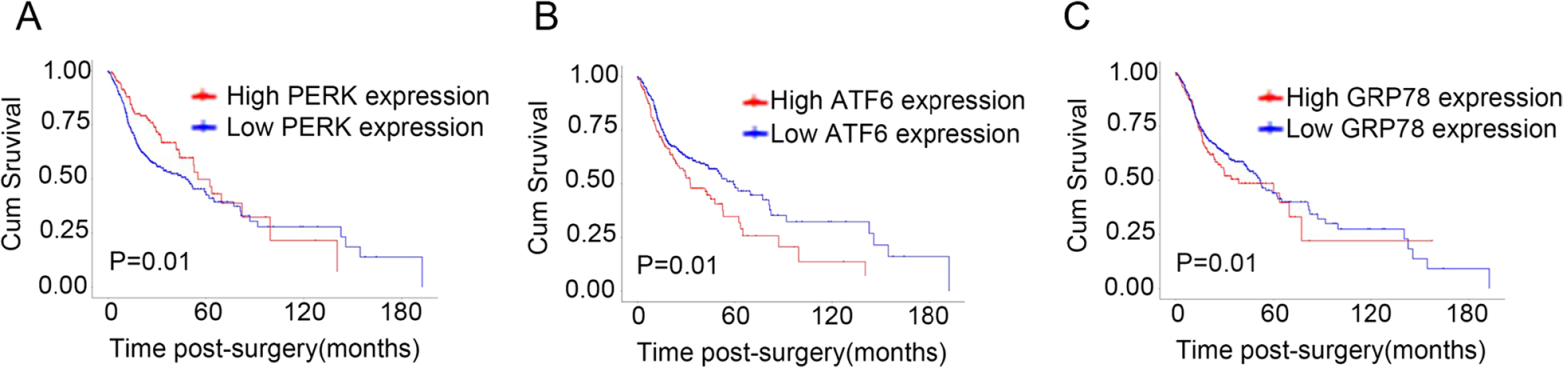
The activation of ER stress correlates with poor survival rates of OSCC patients. The Kaplan–Meier curves indicated significantly shorter overall survival for patients with an overexpression (n = 129) of PERK (**A**), ATF6 (**B**), and GRP78 (**C**) than for those with low expression levels (n = 320) (*P* = 0.01)

To assess the effect of ER stress on OSCC progression, whether ER stress is upregulated among patients with OSCC was tested by the expression measurement of ER stress-associated proteins in 3 cases in which OSCC tissues had been surgically resected using immunohistochemistry. These ER stress-associated proteins contain PERK, ATF6, and GRP78 [20]. As depicted in Fig. 1, the expression of these proteins was much greater in tumor tissues than in matched paracarcinoma tissues (Fig. 1A–D). In addition, a western blot analysis was conducted to determine the ER stress-associated protein expression in three freshly resected OSCC tissues, indicating that the expression of the ER stress-related proteins was much higher in the tumor tissues than in the paracarcinoma tissues (Fig. 1E–H). The qRT-PCR analysis also revealed a higher expression of PERK, ATF6, and GRP78 mRNAs in the tumor tissues than in the paracarcinoma tissues (Fig. 1I–K). Additional file 1: Table S2 displays the clinicopathological characteristics of patients with OSCC. Finally, the association between PERK, ATF6, and GRP78 expression and overall survival was examined for 129 patients with OSCC. According to Fig. 2, the total survival of patients with a low expression of ER stress-associated proteins was greatly higher than that of patients with a high expression of these proteins.

### ER stress-associated protein levels relate to macrophage infiltration and PD-L1 expression in OSCC patients

**Fig. 3:**
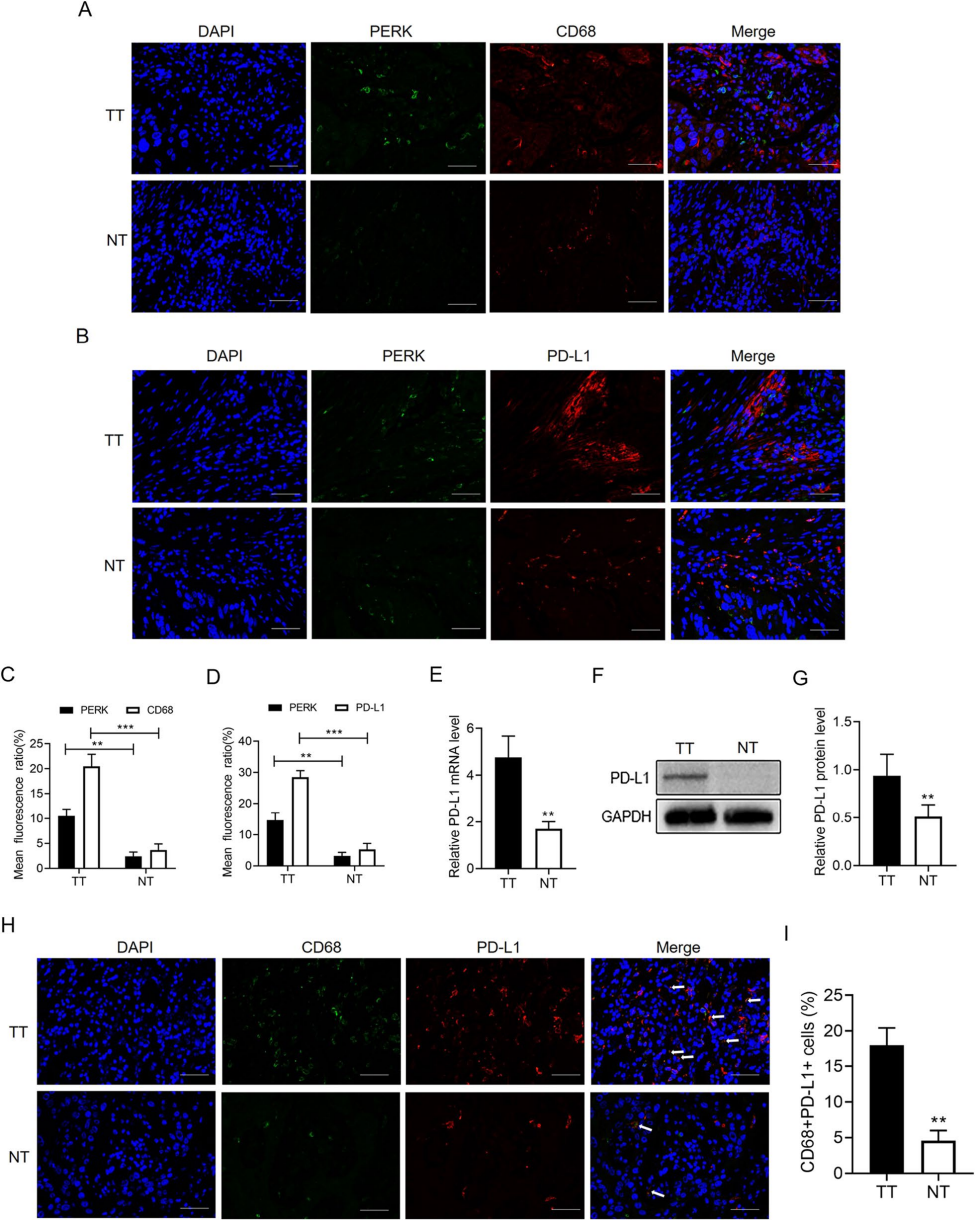
ER stress is associated with macrophage infiltration and PD-L1 expression in OSCC patients. **A** Typical immunofluorescence images of PERK-positive cells and CD68-positive macrophages in the paraneoplastic tissues (NT) and OSCC tissues (TT) of patients with OSCC (scale bar = 50 μm) (n = 2–3). **B** Immunofluorescence staining of PERK-positive cells and PD-L1-positive cells in paraneoplastic tissues (NT) and OSCC tissues (TT) of patients with OSCC (scale bar = 50 μm) (n = 2–3). **C** and **D** Quantification of PERK-positive, CD68-positive, and PD-L1-positive staining in (**A**) and (**B**). **E** The mRNA level of PD-L1 in the paraneoplastic tissues (NT) and OSCC tissues (TT) of patients with OSCC (n = 3). **F** The level of PD-L1 protein in the paraneoplastic tissues (NT) and OSCC tissues (TT) of patients with OSCC. **G** Quantitative analysis of PD-L1 protein levels in (**F**) (n = 3–6). **H** The expression pattern of PD-L1 on CD68 + cells in the paraneoplastic tissues (NT) and OSCC tissues (TT) was measured by immunofluorescence (scale bar = 50 μm) (n = 2–3). **I** Quantification of CD68 + PD-L1 + cell staining in (H) (**P < 0.01 and ***P < 0.001)

It has been shown that ER stress exerts a significant effect on controlling immune cell roles in the tumor microenvironment and antitumor immune responses [21]. Therefore, whether the expression of ER stress-associated proteins including PERK, correlates with macrophage infiltration among patients with OSCC was tested. According to Fig. 3A, the tumor tissues expressing a higher extent of PERK had massive CD68 staining, whereas the CD68+ macrophage infiltration was greatly lower in normal tissues expressing a low extent of PERK. A quantitative exploration displayed that the number of CD68+ regions in the tumor stroma was greatly higher than that in the normal stroma (Fig. 3C).

It was disclosed that PD-L1 stops T cell activation and makes contributions to tumor immune avoidance [22]. To investigate whether ER stress activation relates to PD-L1 expression, an immunofluorescence analysis was adopted to measure the PD-L1 levels, and in the tumor tissues, PD-L1 was often distributed in a crummy way, whereas it only existed sporadically in the normal tissues (Fig. 3B). According to a quantitative analysis, 28.83% of the tumor tissues expressed PD-L1, only 7.17% of the normal tissue expressed PD-L1, and PD-L1 expression positively related to the PERK levels (Fig. 3D). The qRT-PCR and western blot analyses also displayed that the PD-L1 mRNA (Fig. 3E) and proteins (Fig. 3F, G) were markedly upregulated in the tumor tissues as compared to the paired paracarcinoma tissues. To verify whether the infiltrated macrophages expressed PD-L1, immunofluorescence double staining showed that PD-L1 was colocalized with CD68+ macrophages in OSCC tumor stroma (Fig. 3H) frequently. A quantitative exploration revealed that there were 18.02% CD68+PD-L1+ macrophages in OSCC tissues and only 4.57% CD68+PD-L1+ macrophages in normal tissues. According to these outcomes, the ER stress-associated protein levels relate to macrophage infiltration and PD-L1 expression in the OSCC tissues of patients with OSCC.

### Exosomes from ER-stressed OSCC cells upregulate PD-L1 expression in macrophages in vitro

**Fig. 4:**
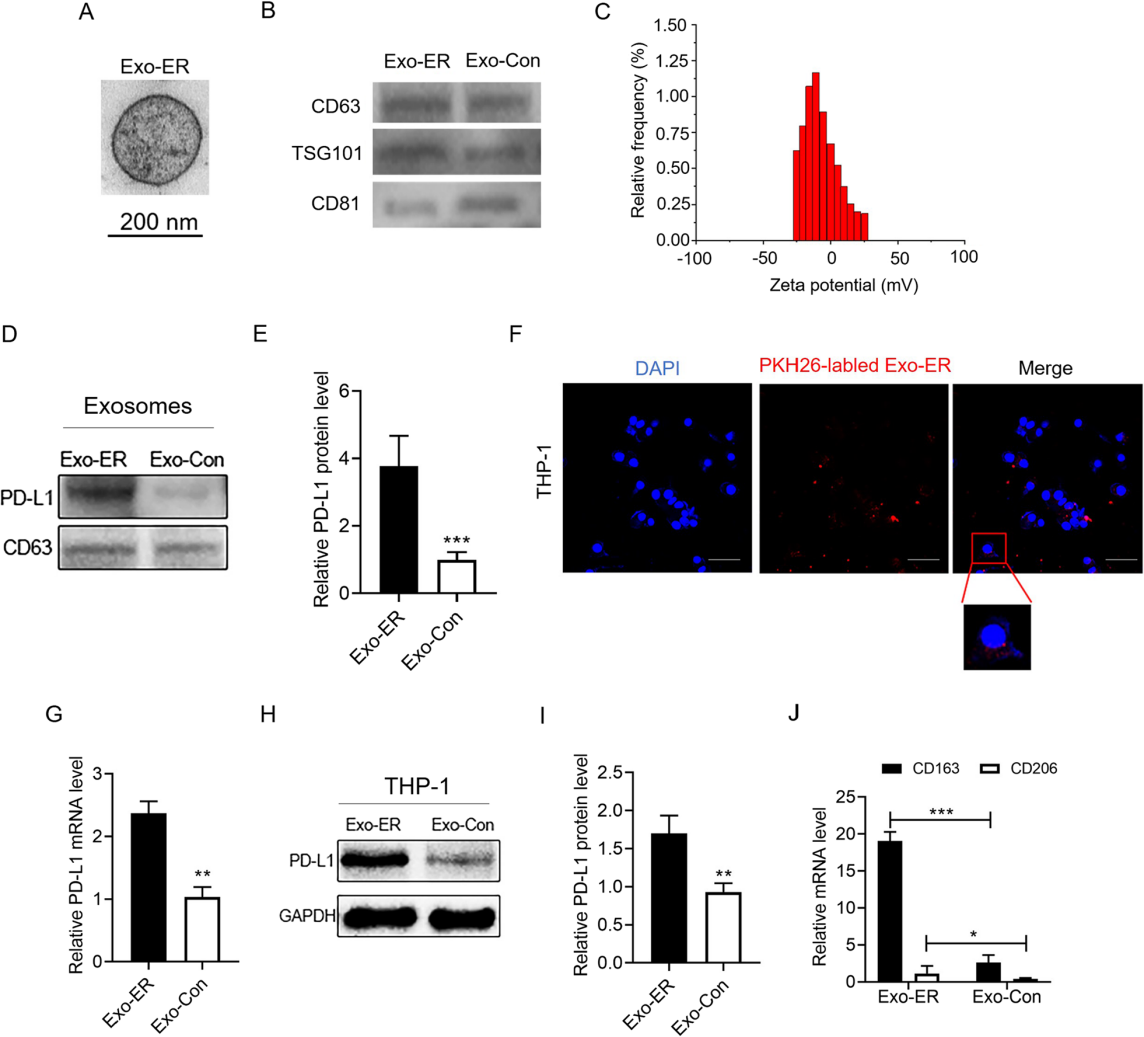
The exosomes from ER-stressed OSCC cells upregulate macrophage PD-L1 expression and induce macrophage polarization toward the M2 subtype in vitro. **A** TEM image of the exosomes isolated from IFN-γ-stimulated (ER-stressed model) HN4 cells (Exo-ER) (scale = 200 nm). **B** Equal amounts of proteins from the exosomes secreted by IFN-γ-stimulated (Exo-ER) or untreated HN4 cells (Exo-Con) were analyzed using western blotting for exosome-enriched protein CD63, TSG101, and CD81. **C** Zeta potential measurements for the Exo-ER. **D** PD-L1 protein levels in Exo-ER and Exo-Con. **E** Quantification of the PD-L1 protein levels in panel (**D**). **F** PKH26-labeled Exo-ER were incubated with THP-1 macrophages and examined using confocal microscopy (scale bar = 50 μm), and the representative images are shown. **G**–**I** The PD-L1 mRNA and protein levels were detected using qRT-PCR (**G**) and western blot (**H** and **I**) analyses in THP-1 macrophages incubated with Exo-ER and Exo-Con. **J** The M2 macrophage markers were detected using qRT-PCR in the THP-1 macrophages incubated with Exo-ER and Exo-Con (n = 2–3) (*P < 0.05, **P < 0.01, and ***P < 0.001)

According to the above information, the occurrence of ER stress is related to upregulated PD-L1 expression in macrophages for OSCC patients, which suggests that ER-stressed OSCCs may drive PD-L1 expression in macrophages. As exosomes function as an important communicator between various cell types, it was assumed that ER-stressed OSCC cells may release exosomes and upregulate PD-L1 expression in macrophages subsequently. To verify this assumption, the *in vitro* cell culture system was optimized for mimiking the ER stress status using interferon-γ (IFN-γ) treatment (500 U/ml for 48 h), which generated a considerable amount of ER stress in the HN4 OSCC cell line. As shown in Fig. 4A, the purity of the exosomes derived from IFN-γ-treated (Exo-ER) HN4 cells was confirmed using TEM, which revealed a homogeneous population of rounded membrane-bound vesicles with a diameter of 30–200 nm. The western blot analysis demonstrated the expression of the exosomal marker protein CD63/TSG101/CD81 (Fig. 4B), and the zeta potential confirmed the stability of the exosome particles in suspension (Fig. 4C). To be interesting, it was observed that the expression of the PD-L1 protein in Exo-ER is higher than that in exosomes originated from untreated HN4 cells (Exo-Con) (Fig. 4D, E). In addition, the co-culture of macrophages with PKH26-marked exosomes showed that THP-1 cells efficiently engulfed the exosomes (Fig. 4F). Further, the qRT-PCR analyses displayed that the upregulation of PD-L1 expression in the THP-1 cells was significantly greater following the co-culture with HN4-derived Exo-ER than that with Exo-Con (Fig. 4G). This PD-L1 upregulation in the macrophages by Exo-ER was also decided by western blotting (Fig. 4H, I). Importantly, treatment with up to 500 U/mL of IFN-γ did not upregulate the PD-L1 levels in the macrophages (Additional file 1: Fig. S1A–C), thereby confirming that the Exo-ER-induced ER stress in macrophages was not a result of IFN-γ contamination.

It was reported that macrophage (predominantly M2 macrophage) infiltration into the tumor microenvironment promotes tumor progression by injuring the immune responses of cytotoxic CD8+ T cells [23]. To determine whether the ER-stressed OSCC cells transmitted PD-L1 to the macrophages and polarized them to the M2 phenotype, the markers of the M2 macrophages were explored. According to Fig. 4J, the coculture with Exo-ER greatly grew the levels of M2 marker (incuding CD163 and CD206) as compared to the Exo-Con treatment. Collectively, these results indicated that ER stress-related exosomal PD-L1 polarized macrophages toward the M2 phenotype.

### Exosomes from ER-stressed OSCC cells upregulate PD-L1 expression in macrophages and induce macrophages to polarize toward the M2 subtype in vivo

**Fig. 5:**
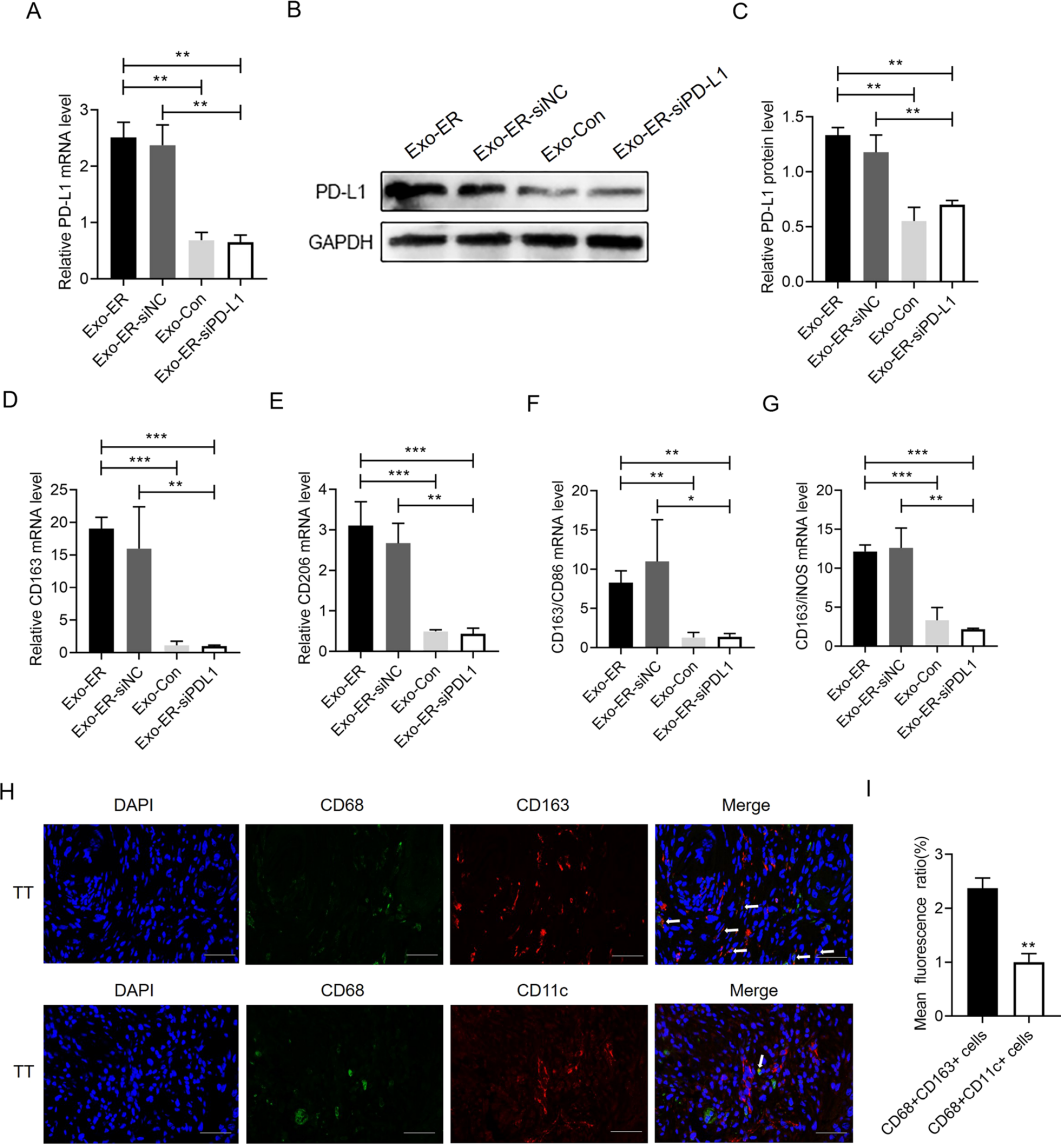
The Exo-ER upregulate the macrophage PD-L1 expression and induce macrophage polarization toward the M2 subtype in vivo. Exosomes were isolated from the culture media of HN4 cells treated with IFN-γ, PD-L1 siRNA (Exo-ER-siPD-L1), or NC siRNA (Exo-ER-siNC). Then, the Exo-ER, Exo-Con, Exo-ER-siNC, or Exo-ER-siPD-L1 (30 μg/mouse) were injected into six-week-old female nude mice through the tail vein. The peritoneal macrophages were isolated, and the expression of PD-L1 was evaluated using the qRT-PCR (**A**) and western blot (**B**) analyses (n = 2–3). **C** Quantification of PD-L1 protein levels in panel (**B**). **D** and **E** Relative CD163 (**D**) and CD206 M2 macrophage markers (**E**) mRNA expression in the isolated peritoneal macrophages (n = 3). **F** and **G** The ratio of M2 macrophage markers (CD163) and M1 macrophage markers (CD86 and iNOS) was shown in the isolated peritoneal macrophages (n = 3). **H** Immunofluorescence staining of CD68, CD163, and CD11c in OSCC tissues (TT) of patients with OSCC (scale bar = 50 μm) (n = 2–3). **I** Quantification of the CD68 + CD163 + cells (M2 macrophages) and CD68 + CD11c + cells (M1 macrophages) in (**A**) (*P < 0.05, **P < 0.01, and ***P < 0.001)

To decide whether Exo-ER also enhances PD-L1 expression *in vivo*, nude mice were injected with Exo-ER, Exo-Con, Exo-ER-siPD-L1, or Exo-ER-siNC once every two days for a total of 10 times, and the peritoneal macrophages were isolated at 12 h after the last injection. As illustrated in Additional file 1: Fig. S2A–C, PD-L1 expression was decreased in HN4 cells treated with siPD-L1, but not in the HN4 cells treated with the siNC. In line with this, knockdown of PD-L1 in HN4 cells inhibited the PD-L1 expression in exosomes secreted by HN4 cells (Additional file  1: Fig. S2D–E). Furthermore, the qRT-PCR and western blot analyses demonstrated that the levels of PD-L1 were greatly higher in the peritoneal macrophages separated from the Exo-ER-injected mice than those from the Exo-Con and Exo-ER-siPD-L1 groups (Fig. 5A–C). Further, the peritoneal macrophages separated from the Exo-ER-siNC-injected mice also presented greatly higher levels of PD-L1 by comparing with the Exo-ER-siPD-L1 group (Fig. 5A–C). Besides, these macrophages also had higher levels of M2 markers (such as CD163 and CD206) and M2/M1 ratio (such as CD163/CD86 and CD163/iNOS) compared to those from the Exo-Con or Exo-ER-siPD-L1 groups (Fig. 5D–G). More interestingly, immunofluorescence double staining was conducted, and the tumor tissue-accumulated macrophages were CD68+CD163+ macrophages (M2-polarized macrophages) (Fig. 5H, I), indicating a greater importance of the M2 subtype in OSCC progression. Collectively, these results suggested that ER stress can upregulate the expression of PD-L1 in HN4-derived exosomes. The exosomes can transfer PD-L1 from the ER stress cancer cells to the macrophages and upregulate PD-L1 expression in the same. PD-L1 could also accelerate macrophage polarization toward the M2 subtype.

## Discussion

According to many researches, antitumor immunity through different mechanisms can be dysregulated by ER stress, including the stimulation of pro-tumor inflammatory factors and the induction of MDSCs [12, 15, 24]. The current research recognized a novel mechanism by which ER stress promotes the release of PD-L1-enriched exosomes by OSCC cells. These exosomes then up-regulate PD-L1 expression by the macrophages and promote the polarization of the macrophages toward the M2 subtype accordingly. Most of the human OSCC samples examined here showed a high expression of ER stress-associated proteins, and their expression levels were negatively related to the patients’ overall survival. The tumors also had a greater number of CD163+ macrophages and PD-L1+ cells in the stroma, and the PD-L1 expression was also related to poor overall survival of OSCC patients. We also found that ER-stressed OSCC cells upregulated macrophage PD-L1 expression and promoted M2 macrophage differentiation accordingly through the transmission of exosomal PD-L1, leading to the elevation of PD-L1 levels. Combined, our results indicate that OSCC cells can transmit ER stress signals to infiltrating macrophages through the release of PD-L1-enriched exosomes, thereby promoting tumor progression.

ER stress promotes the release of exosomes from OSCC cells and modulates PD-L1 expression in macrophages. It has been revealed that immune escape of tumor cells is promoted by establishing an immunosuppressive microenvironment, but the exact underlying mechanism is unknown. It has been reported that exosomes exert an important effect on intercellular communication by conveying their contents (including proteins) to the “recipient” cells [25, 26]. For instance, Kanemoto et al. displayed that ER stress drove exosome release in a PERK-dependent manner [27]. However, the role of the exosomes released by ER-stressed OSCC cells in immune cell function and the potential immunomodulation by specific exosomal proteins are not clear.

Recently, according to a number of researches, exosomal PD-L1 plays an important role in tumor progression and evasion [19, 28], and we found that the exosomes from ER-stressed OSCC cells have a high level of PD-L1. Macrophages are the predominant cells in the tumor stroma, and a high macrophage density has been related to a distasteful prognosis in various kinds of tumors [24]. Some researches have revealed that macrophages promote tumor progression through direct communication with the tumor cells [29]. We found that OSCC tissues hired more CD68+ macrophages into the stroma than was observed in normal tissues. These exosomes were also moved into macrophages effectively and transmitted ER stress signals to the same, indicating that ER stress facilitates OSCC cell exosome release and the transmission of PD-L1-enriched exosomes to the macrophages located in the tumor microenvironment.

Interestingly, we also found that overexpression of PD-L1 in THP-1 macrophages induced CD206 expression in THP-1 cells (Additional file 1: Fig. S3), which implied that macrophages expressing high levels of PD-L1 polarize toward the M2 subtype. However, the mechanism by which PD-L1 promoted M2 polarization in macrophages should be carried out in future. PD-L1, also known as B7-H1 or CD274, inhibits T cell proliferation and drives T cell dysfunction by binding to programmed death 1 (PD-1) proteins. Hematopoietic and nonhematopoietic cells are used to express PD-L1, and the upregulation of PD-L1 has been found in different tumors, where it makes contributions to immune evasion. There are high extents of PD-L1 in macrophages, where the PD-L1 suppresses antitumor immunity [30–32]. It has been reported that macrophage infiltration into the tumor microenvironment promotes tumor progression by impairing the immune responses of cytotoxic CD8+ T cells. Besides, blocking PD-L1 with a given antibody enhanced the T cell immune response, indicating that growing PD-L1 expression in macrophages assists OSCC cells in escaping from cytotoxic T cells. PD-L1 can prevent T cell activation and makes contributions to tumor immune evasion in many cancers, such as liver cancer and mammary cancer. In addition, macrophages that infiltrate into liver cancers can express higher levels of PD-L1; therefore, we assumed that the same trend would occur in OSCC [33–35].

This research displayed that the macrophages that infiltrated into ER-stressed OSCC tissues expressed high levels of PD-L1 and that the PD-L1 levels were negatively related to total survival. The macrophages are classified into M1 and M2 macrophages: M1 macrophages exert a protective effect, whereas M2 macrophages drive tumor development [36, 37]. Our *in vitro* and *in vivo* researches displayed that Exo-ER polarized macrophages to the M2 phenotype, indicating that ER-stressed OSCC cells may “teach” macrophages to assume the M2 subtype, thereby facilitating disease progression. Our results also indicated that ER stress promotes OSCC cells to release PD-L1-strengthened exosomes and that these exosome-treated macrophages greatly upregulate PD-L1 expression and polarize toward the M2 subtype to promote tumor growth.

## Conclusion

The current study provides evidence indicating that the exosomal PD-L1 released by ER-stressed OSCC leads to immunosuppression by inducing M2 macrophage polarization. Further, our findings provide a foundation for ER-stressed OSCC exosomes research aimed at obtaining a greater understanding of their pathological importance. In addition, the delineation of a new exosomal-modulated mechanism was made for OSCC–macrophage crosstalk promoting tumor development and should be dug for its therapeutic utility.

## Supplementary Information

**Additional file 1**.** Fig. S1**. IFN-γ did not affect PD-L1 expression in the treatment macrophages. (A) The THP-1 macrophages were treated with Exo-ER, IFN-γ, or PBS (Blank), followed by the detection of the PD-L1 mRNA (A) and protein levels (B and C) (n = 2-3) (*P < 0.05 and **P < 0.01; NS, no significance).** Fig. S2**. Knockdown of PD-L1 in HN4 cells and HN4-derived exosomes. HN4 cells were transfected with siPD-L1 or siNC, and exosomes were collected. (A-C) PD-L1 mRNA and protein level were detected in treatment HN4 cells by qRT-PCR (A) and Western blot (B and C). (D-E) Exosomal PD-L1 protein level was detected in treatment HN4 cells (n = 2-3) (*P < 0.05 and **P < 0.01).** Fig. S3**. Overexpression of PD-L1 promoted M2 polarization in macrophages. qRT-PCR analysis of CD206 mRNA in THP-1 macrophages transfected with plasmid encoding control (OE-NC) or PD-L1 (OE-PD-L1) (n = 3) (**P < 0.01).** Table S1**. RNA sequence used in this paper.** Table S2**. Clinicopathologic features of OSCC patients.

## Data Availability

The datasets generated during and/or analyzed during the current study are available from the corresponding author on reasonable request.

## Abbreviations

OSCC: Oral squamous cell carcinoma
TME: Tumor associated environment
ER: Endoplasmic reticulum
PERK: Protein kinase R-like ER kinase
ATF6: Activating transcription factor 6
GRP78: Glucose-regulated protein 78
IRE1α: Inositol-requiring enzyme 1α
Exo: Exosomes
Exo-Con: Exosomes isolated from untreated HN4 cells
Exo-ER: Exosomes isolated from IFN-γ–treated HN4 cells
Exo-ER-siNC: Exosomes isolated from IFN-γ and NC siRNA-treated HN4 cells
Exo-ER-siPD-L1: Exosomes isolated from IFN-γ and PD-L1 siRNA-treated HN4 cells
MDSCs: Myeloid-derived suppressor cells
TAMs: Tumor-associated macrophages
TILs: Tumor-infiltrating T cells
WHO: World Health Organization
TNM: Tumor-node-metastasis
PCR: Polymerase chain reaction
TEM: Transmission electron microscopy
IFN-γ: Interferon-γ
PD-L1: Programmed cell death-ligand 1
