# Supplementary material for: Endoplasmic reticulum stress promotes the release of exosomal PD-L1 from head and neck cancer cells and facilitates M2 macrophage polarization
Source: Cell Commun Signal. 2022 Jan 28;20:12. doi: 10.1186/s12964-021-00810-2 (PMC8796490; doi:10.1186/s12964-021-00810-2)
Supplement: Supplementary file 2 — Additional file 1. Fig. S1. IFN-γ did not affect PD-L1 expression in the treatment macrophages. (A) The THP-1 macrophages were treated with Exo-ER, IFN-γ, or PBS (Blank), followed by the detection of the PD-L1 mRNA (A) and protein levels (B and C) (n = 2-3) (*P < 0.05 and **P < 0.01; NS, no significance). Fig. S2. Knockdown of PD-L1 in HN4 cells and HN4-derived exosomes. HN4 cells were transfected with siPD-L1 or siNC, and exosomes were collected. (A-C) PD-L1 mRNA and protein level were detected in treatment HN4 cells by qRT-PCR (A) and Western blot (B and C). (D-E) Exosomal PD-L1 protein level was detected in treatment HN4 cells (n = 2-3) (*P < 0.05 and **P < 0.01). Fig. S3. Overexpression of PD-L1 promoted M2 polarization in macrophages. qRT-PCR analysis of CD206 mRNA in THP-1 macrophages transfected with plasmid encoding control (OE-NC) or PD-L1 (OE-PD-L1) (n = 3) (**P < 0.01). Table S1. RNA sequence used in this paper. Table S2. Clinicopathologic features of OSCC patients. [file 12964_2021_810_MOESM2_ESM.docx]

**Supplymental data**


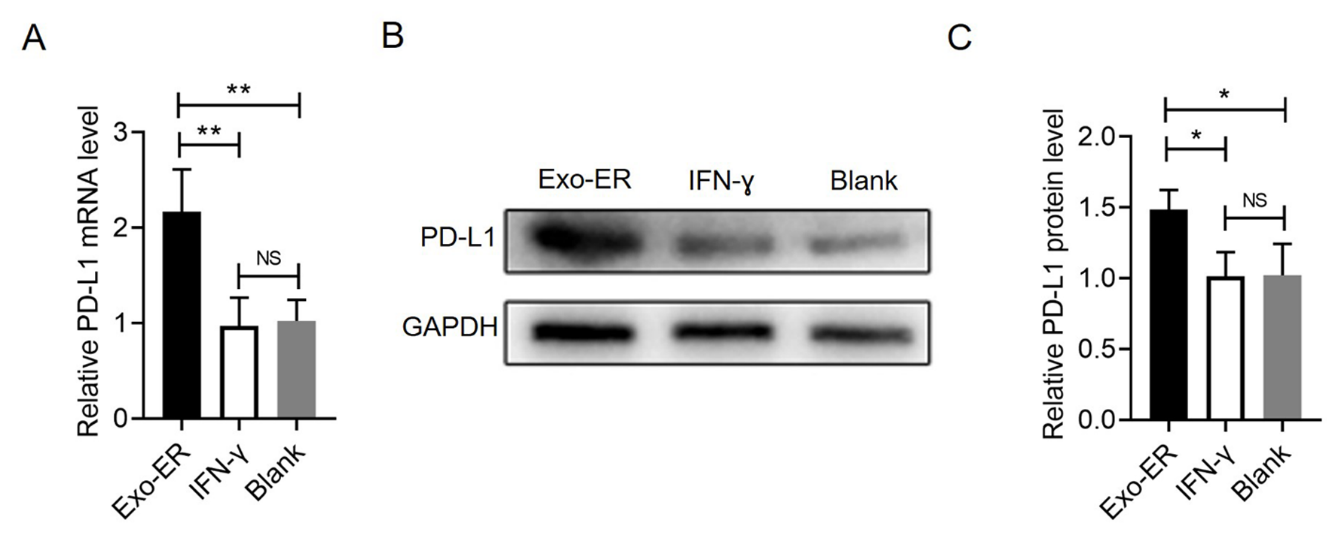


**Fig. S1** IFN-γ did not affect PD-L1 expression in the treatment macrophages. (A) The THP-1 macrophages were treated with Exo-ER, IFN-γ, or PBS (Blank), followed by the detection of the PD-L1 mRNA (A) and protein levels (B and C) (n = 2-3) (*P < 0.05 and **P < 0.01; NS, no significance).

**
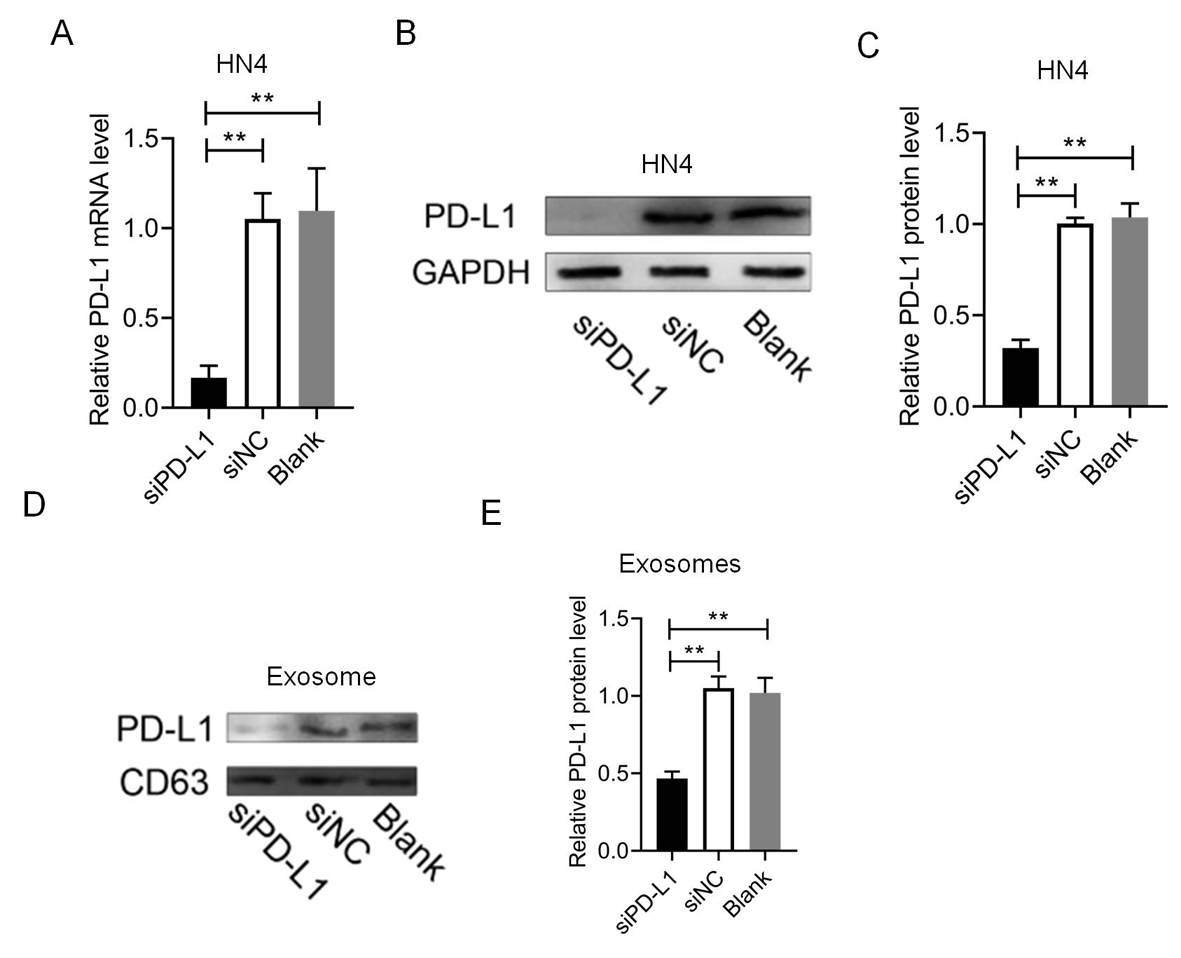
**

### Fig. S2 Knockdown of PD-L1 in HN4 cells and HN4-derived exosomes. HN4 cells were transfected with siPD-L1 or siNC, and exosomes were collected. (A-C) PD-L1 mRNA and protein level were detected in treatment HN4 cells by qRT-PCR (A) and Western blot (B and C). (D-E) Exosomal PD-L1 protein level was detected in treatment HN4 cells (n = 2-3) (*P < 0.05 and **P < 0.01).

**
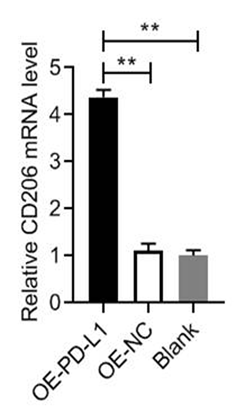
**

**Fig. S3** Overexpression of PD-L1 promoted M2 polarization in macrophages. [qRT-PCR analysis](https://www.sciencedirect.com/topics/biochemistry-genetics-and-molecular-biology/western-blot) of CD206 mRNA in THP-1 macrophages transfected with [plasmid](https://www.sciencedirect.com/topics/biochemistry-genetics-and-molecular-biology/lentiviruses) encoding control (OE-NC) or PD-L1 (OE-PD-L1) (n = 3) (**P < 0.01).

**Table S1** RNA sequence used in this paper.

|  | Primer (5'-3') |
| --- | --- |
| Human PERK | F: GTCCCAAGGCTTTGGAATCTGTC |
|  | R: CCTACCAAGACAGGAGTTCTGG |
| Human ATF6 | F: CAGACAGTACCAACGCTTATGCC |
|  | R: GCAGAACTCCAGGTGCTTGAAG |
| Human GRP78 | F: CTGTCCAGGCTGGTGTGCTCT |
|  | R: CTTGGTAGGCACCACTGTGTTC |
| Human PD-L1 | F: TGCCGACTACAAGCGAATTACTG |
|  | R: CTGCTTGTCCAGATGACTTCGG |
| Human CD163 | F: CCAGAAGGAACTTGTAGCCACAG |
|  | R: CAGGCACCAAGCGTTTTGAGCT |
| Human CD206 | F: AGCCAACACCAGCTCCTCAAGA |
|  | R: CAAAACGCTCGCGCATTGTCCA |
| Human CD86 | F: CCATCAGCTTGTCTGTTTCATTCC |
|  | R: GCTGTAATCCAAGGAATGTGGTC |
| Human iNOS | F: GCTCTACACCTCCAATGTGACC |
|  | R: CTGCCGAGATTTGAGCCTCATG |
| Human GAPDH | F: GTCTCCTCTGACTTCAACAGCG |
|  | R: ACCACCCTGTTGCTGTAGCCAA |

**Table S2** Clinicopathologic features of OSCC patients.

| Clinicopathologic features | Case (n) |
| --- | --- |
| Age (years) |  |
| <60 | 41 |
| >=60 | 59 |
| Gender |  |
| male | 61 |
| female | 39 |
| Stage |  |
| 1 | 17 |
| 2 | 36 |
| 3 | 29 |
| 4 | 18 |
| Lymph node metastasis |  |
| negative | 53 |
| positive | 45 |
| unknown | 2 |
| Grade |  |
| well differentiated | 47 |
| moderate differentiated | 37 |
| poor differentiaed | 16 |
| Smoking |  |
| no | 47 |
| yes | 53 |
| Alcohol |  |
| no | 51 |
| yes | 49 |
| Recurrence |  |
| no | 84 |
| yes | 16 |
